# Participation in Physical Activity for Children with Neurodevelopmental Disorders

**DOI:** 10.1155/2012/460384

**Published:** 2012-04-30

**Authors:** Rubab G. Arim, Leanne C. Findlay, Dafna E. Kohen

**Affiliations:** Health Analysis Division, Statistics Canada, RH Coats 24, 100 Tunney's Pasture Way, Ottawa, Ontario, Canada K1A 0T6

## Abstract

The purpose of this study was to compare rates of participation for children (4–9 years of age) with neurodevelopmental disorders (NDDs) with and without externalizing behavior problems (EBPs) with children without disability and to examine mediators of the relation between disability and physical activity participation. Data for this study were drawn from Cycle 7 (2006-07) of the Canadian National Longitudinal Survey of Children and Youth (NLSCY). The frequency of children's participation in organized sports or physical activities varied depending on the child's health condition with children with NDDs and both NDDs and EBPs participating least in organized sports or physical activities followed by children with EBPs only. In contrast, there were no statistically significant differences by health group for children's participation in unorganized sports or physical activities. These differences remained even after controlling for the effects of other child and family sociodemographic characteristics, except for children with EBPs only. These findings highlight the importance of considering children's primary and other existing health conditions as well as family sociodemographic characteristics in order to better understand the factors that influence participation in organized physical activities for children with disabilities.

## 1. Introduction

The advantages of participation in physical activity for children are well established, including physical, psychosocial, and emotional benefits [1, 2]. For children with disabilities who may experience impairments in mobility, functioning, and overall well-being, participation in physical activity may be particularly valuable in that physical activity promotes physical development and health within a social context [3]. However, children with disabilities may be limited in terms of motor abilities and social skills, thus impacting their ability to participate in physical activities. Despite this, specific physical activity recommendations have been made by the Canadian Paedeatric Society [4] for certain chronic disease conditions including juvenile idiopathic arthritis, hemophilia, asthma, and cystic fibrosis, pointing to the recognition or importance of physical activity for children with chronic health conditions or disabilities. The purpose of the current study is to compare rates of participation for children with neurodevelopmental disorders with and without externalizing behavior problems with children without disability, and to examine mediators of the relation between disability and activity participation. Imms et al. [5] and King et al. [6] found disparate correlates of informal (unorganized) versus formal (organized) physical activity for children with disabilities, thus these two types of activities were considered separately.

Reviews of the physical activity and disability literature suggest that participation in physical activity is beneficial for children with disabilities for therapeutic reasons as well as general physical and social development [7]. Although disease state and individual capacity must be considered, the potential benefits of physical activity participation (including psychosocial, muscular strength, and cardiovascular capacity) may offset or even counteract disease progression, for example, for children with asthma or juvenile idiopathic arthritis [8]. Physical activity participation might also be advantageous in minimizing or counteracting secondary health (e.g., diabetes, obesity) and impairments (e.g., decreased strength, poor balance) for children with chronic health conditions [4, 9–11]. Finally, in addition to the physical benefits, physical activity participation has been associated with enhanced social identity, including perceptions of competence and similarity to peers, enhanced self-worth, and strengthening social interaction and bonding [3].

An NDD is an impairment of the growth and development of the central nervous system, emerging in early development [12] and impacting various domains of functioning, including ambulation, information-processing, self-regulation, and communication [13]. Examples of NDDs include cerebral palsy, intellectual disability, and autism spectrum disorder. In Canada, 6% of children between the ages of 4 to 11 years were found to have an NDD [14]. Children with an NDD are also at greater risk of developing emotional and behavioral problems compared to their peers [15, 16]. Over half (55%) of Canadian children with an NDD were also found to exhibit one or more externalizing behavior problems (EBPs) [14]. EBPs refer to a group of behavior problems that are manifested in children's outward behavior [17] such as conduct disorder/physical aggression, indirect aggression, and hyperactivity-inattention problems.

While an abundance of information is available regarding rates of physical activity participation for children in general [1, 18, 19], less is known about rates of participation for children with disabilities specifically. Children with disabilities are less likely to engage in physical activity than their healthy peers [4, 10, 11, 20] and much of the literature on physical activity and disability has focused on children with cerebral palsy (CP) as it is the most common form of childhood disability [11, 21–23]. Bjornson and colleagues [21] found that children aged 8 to 13 years with CP took fewer steps per day (as measured by accelerometry) and were less active overall than typically developing peers. These differences were attenuated by gross motor ability, such that the highest functioning individuals (according to the Gross Motor Function Classification System, GMFCS [24]) were more similar to children with typical development [21].

Using data from a national Canadian disability survey, Kowalchuk and Crompton [25] found that 63% of Canadian children with disabilities (those who had difficulties with daily living activities or those for whom a physical or mental condition or health problem reduced activities) engaged in organized sport or physical activity, most of whom were doing so at least once per week. These results are higher than those reported by Longmuir and Bar-Or [26] who found that within their clinical sample of Canadian children, 47% of those with a chronic medical condition (e.g., kidney disease, hearing impairment) and 26% of those with a physical disability (including CP, brain injury, spina bifida, and muscular dystrophy) were physically active. Unfortunately, this study did not include a control/comparison group. Differences in these reported rates of participation are likely due to differences in the sample characteristics (e.g., the age of the study participants), the identification of disabilities, and broad definitions of physical activity. For instance, in Kowalchuk and Crompton's study, children were considered to be active (versus inactive) if they had participated in any organized sport or activity in the past 12 months, regardless of frequency, whereas Longmuir and Bar-Or created three categories of activity (active, moderately active, and sedentary) based on frequency, intensity, and duration of activities.

Children with comorbid conditions such as a physical disability and communication deficits may be at further risk of reduced physical activity participation [27]. This would suggest that research considers not only the complexity of the individual's medical or social impairment, but also any additional conditions which may impede participation in physical activity. While the general literature on EBPs and physical activity has not shown a link between EBPs such as aggression and sports participation [28, 29], to our knowledge, there are no existing studies that have investigated physical activity participation for children with NDDs with and without EBPs.

In addition to a lower amount of physical activity, children with disabilities tend to partake in different types of activities, particularly lower intensity activities, than their healthy peers [20, 30]. Despite recent advances in specific programs such as Special Olympics or Paralympic Games, children with physical disabilities may have fewer opportunities to engage in formal, organized activities, in particular competitive or elite sports contexts [3]. Some evidence has suggested that children with disabilities participate in predominantly informal, home-based activities [23, 31, 32] and are less inclined to participate in physical-based leisure activities as compared to social, recreational, or self-improvement type activities [22].

While much is known regarding the barriers and facilitating conditions of physical activity for children and youth in general [33], relatively few studies have examined barriers or facilitators to participation in physical activity for children with disabilities [23, 32, 34]. The identification of such factors may help inform recommendations for identifying resources and/or strategies for increasing opportunities and rates of participation for children with disabilities. For example, previous work on children with CP specifically has suggested that low parental education [11] and lower parental income [32] are associated with less participation in physical activity. Physical ability, often measured as gross motor function, has also been associated with participation such that better motor function has been associated with greater active-physical leisure time activity [5, 20, 23]. Similarly, Longmuir and Bar-Or [26] found that physical activity level varied dramatically by disability type, with those having more severe impairments being less likely to participate than those with less severe impairments. Moreover, Kowalchuk and Crompton [25] found that participation in physical activity was more likely if the child had a physical (70%) or nonphysical (69%) disability (e.g., learning disability), as compared to both physical and nonphysical (59%), and if the severity was mild (70%) as compared to very severe (45%). However, these differences in physical activity participation did not remain statistically significant once sociodemographic correlates were considered, suggesting that child or family characteristics (including child age, family income, and barriers to participation) were stronger determinants of participation than type or severity of disability. Identification of any comorbid conditions is thus an important consideration.

Other characteristics have also been examined as potential correlates of participation. Unlike findings in the typical child development literature whereby boys are more likely to engage in physical activity than are girls [33, 35], an effect of gender has not typically been shown for participation in physical activity for children with disabilities [5, 6, 11, 20, 22, 26]. In one study, however, Law and colleagues [31] found that boys were more likely to participate in active physical activities than were girls (aged 6–14), as were children of dual-parent families as compared to single-parent families. Moreover, age is a potential determinant, with young (i.e., age 5 [11]) and older (age 17 [20]) children being less likely to be active than children in the mid-age range (ages 7 and 11, resp.). In the typical child development literature, access to recreational facilities has also been associated with physical activity participation [36].

Previous research on physical activity for children with disabilities has thus been limited by (i) a focus on children with CP, (ii) inconsistent definitions of physical activity, (iii) lack of control groups (i.e., comparisons to healthy children), and (iv) the omission of other comorbid conditions such as EBPs. The current study examines rates of participation in physical activity for children with NDDs living in Canada. In Canada, children's participation in physical activities often occurs outside of the school setting. The advantages of the present study include the use of a population-based set of data, a noncategorical definition of disability including NDDs with and without EBPs, comparisons with a control group of children without disability, and an examination of both organized and unorganized physical activity participation. Our study seeks to address two questions: (1) Are there differences in participation in organized and/or unorganized physical activity for children with NDDs, with and without EBPs, compared to children without disability?, and (2) What sociodemographic characteristics mediate differences between NDDs, EBPs, both NDDs and EBPs, and children without disability?

## 2. Materials and Methods

### 2.1. Data Source and Sample

Data for this study were drawn from Cycle 7 (2006-07) of the Canadian National Longitudinal Survey of Children and Youth (NLSCY), a longitudinal study of the physical and social development of Canadian children from birth into adulthood. The NLSCY, jointly conducted by Human Resources and Skills Development Canada (HRSDC) and Statistics Canada, started in 1994 and was repeated biennially. The first cohort of children who participated in the survey was between the ages of 0 to 11 years. The person most knowledgeable (PMK) of the child provided information on the participant child as well as information about him/herself, and his/her spouse or partner by completing a series of questionnaires in the household. The surveys were completed using computer- assisted interviewing (CAI) methods (either via personal interviewing in the household or telephone interviewing). In 90% of cases, the PMK was the biological mother of the child. In this study, the PMK will be referred to hereafter as the parent.

In 1994-1995, a total of 22,831 children were proportionally sampled from all areas of the country excluding children living in institutional settings and residing in Yukon, Nunavut, Northwest Territories, and First Nations reserves [37]. The cross-sectional sample for this study consisted of children who were between 4 and 9 years of age in Cycle 7 (2006-07); this was the most recent cycle in which all relevant variables were available.

### 2.2. Measures

#### 2.2.1. Child Health Groups

The classification of child health consisted of four groups: (a) children with an NDD only (NDD), (b) children with an EBP only (EBP), (c) children with both an NDD and an EBP (BOTH), and (d) children who had neither a chronic condition (including an NDD or an EBP) nor an activity limitation (HEALTHY). The presence of an NDD in children was identified using a checklist of chronic conditions diagnosed by a health professional. Four parent-reported chronic conditions, including epilepsy, CP, intellectual disability, and a learning disability, were used for this definition, and 423 (3.4%) children were identified as having an NDD. This definition has been used in previous research [14, 38, 39]. It should be noted that approximately ten children were missing information required to identify the presence of an NDD and were, therefore, excluded from the sample. In addition, on the questionnaire, the term mental handicap was used to represent an intellectual disability.

The presence of an EBP was identified using a parent-reported child behavior rating scale, with items derived from the Child Behavior Checklist (CBCL [40]) and modified for Canadian children. Three EBP scales were used: hyperactivity-inattention (8 items, Cronbach's alpha = .87 [41]; for example, “How often would you say that [child's name] cannot sit still, is restless or hyperactive?”), physical aggression-conduct disorder (6 items, Cronbach's alpha = .89; for example, “How often would you say that [child] gets into many fights?”), and indirect aggression (5 items, Cronbach's alpha = .82; for example, “How often would you say that [child] when mad at someone, tries to get others to dislike that person?”). Each item was scored on a 3-point scale ranging from 0 (*never or not true)* to 2 (*often or very true*). Consistent with our previous work [14, 38], children were considered to have an EBP if at least one of their scores on the three EBP scales was two standard deviations above the mean or greater. A total of 1520 (12.3%) children were identified as having an EBP using these criteria. None of the children in the sample had missing information required to identify an EBP.

To identify children without disability (HEALTHY group), we used two exclusion criteria: (1) presence of another (non-NDD) chronic condition (a long-term condition diagnosed by a health professional, such as a heart condition, asthma, kidney disease, bronchitis, and diabetes), (2) limitation in activities (due to asthma or any other activity limitations at home, at child care, at school, or other settings). There were 3266 children who had a long-term condition and/or activity limitation and thus were excluded from the group of children without disability (HEALTHY group).

After the final classification of child health into four groups (*N* = 9119), there were 286 children in the NDD group, 1382 children in the EBP group, 137 children in the BOTH group, and 7314 children in the HEALTHY group. Approximately 25% of children in the NDD group were reported to have activity limitations at home and almost half of these children were reported to have limitations in “other” activities, including activities at child care or at school, and transportation or sports and games. Only 4.2% of children in the EBP group were found to have activity limitations at home and 8% of them had limitations in “other” activities. The percentages of children with activity limitations were higher in the BOTH group of children compared to the other groups. Approximately 43% of children who had both an NDD and EBP were found to have activity limitations at home and 63% of them were reported to have limitations in “other” settings and activities.

#### 2.2.2. Sociodemographic Characteristics

Two other child characteristics were considered: child age (0 = preschool-age (4-5 years old), 1 = school-age (6–9 years old)), and sex (0 = male; 1 = female). In addition, four family characteristics were included in the analyses: single parent (0 = no; 1 = yes), parent's educational attainment (0 = less than or equal to high school education; 1 = greater than high school education), household income (0 = low income; 1 = moderate income; 2 = high income), and child living in a census metropolitan area (CMA) or census agglomeration (CA) using 2006 Census of Canada code (0 = no; 1 = yes). Living in a CMA or CA was used to identify areas expected to have higher access to services (i.e., higher populated areas).

The measure of household income was based on a comparison between parents' report of an estimate of their household income and the low income cutoff (LICO) score established by Statistics Canada. The LICO indicates an income level at which a family will likely spend a greater portion of its income on basic needs such as food, clothing, and shelter than does an average family of similar size [42]. An income-to-LICO ratio of less than 1 is generally defined as living in low income. In this study, a household income-to-LICO ratio equal to or greater than 1 but less than 2 was defined as moderate income, while an income-to-LICO ratio equal to or greater than 2 was defined as high income.

According to information on 2006 Standard Geographical Classification (SGC) developed by Statistics Canada [43], a CMA is a standard geographical entity consisting of an urban core, including several adjacent urban and rural areas that have a high degree of social and economic integration with that urban core. A CMA must have a population of at least 100,000 with an urban core of 50,000. To form a CA, the urban core must have a population of at least 10,000. It should be noted that if the population of the urban core of a CA declines below 10,000, the CA is retired. However, once an area is defined as a CMA, it is retained as a CMA even if its population declines below 100,000.

#### 2.2.3. Outcome Variables

 Two outcome variables were considered: children's participation in *organized* sports or physical activities and children's participation in *unorganized* sports or physical activities in the last year. The variable on organized sports or physical activities was based on two items from the survey, which asked parents to indicate how often the child had taken part in (a) sports with a coach or instructor (except dance, gymnastics or martial arts) in the past 12 months, and (b) lessons or instruction in other organized physical activities with a coach or instructor such as dance, gymnastics or martial arts in the past 12 months. Response options for both items range from 1 (*most days*) to 5 (*almost never*). Similar to previous research [44], a composite item was created to indicate children's participation in organized sports or physical activities in the last year, which was dichotomized (0 = about once a month or almost never; 1 = about once a week or more) due to skewness in responses. The second outcome variable was also a parent-reported item that assessed how often the child had taken part in unorganized sports or physical activities without a coach or instructor. The response scale was similar to the organized sports or physical activities outcome variable, and for the same reason (i.e., skewness), the responses were dichotomized to represent children's participation in unorganized sports or physical activities at least once a week versus about once a month or almost never.

### 2.3. Data Analysis

First, we calculated descriptive statistics (i.e., percentages) for each variable. Second, we conducted chi-square tests to examine group differences with the reference group being children without disability (i.e., children in the HEALTHY group), including group differences in participation in organized and/or unorganized physical activity for children with NDDs, with and without EBPs, compared to children without disability. The next set of analyses included a series of logistic regressions. In Model 1, we examined the associations between child health group and participation in organized sports or physical activities. In Model 2, we examined these associations after controlling for the effects of other child characteristics (i.e., age and sex). In Model 3, we included family sociodemographic characteristics in addition to the child characteristics and examined whether these factors played a role in explaining the relationships between child health group and participation in organized sports or physical activities. Moreover, we conducted contrast analyses to determine whether there were significant differences in beta coefficients among the child health groups. Finally, we performed additional logistic regression analyses, where we examined the effects of each variable separately, to determine what sociodemographic characteristics mediate differences between NDDs, EBPs, both NDDs and EBPs, and children without disability. All analyses were weighted and bootstrapped to account for complex survey design [45].

## 3. Results

Descriptive statistics for the total sample and for each child health group are presented in Table 1. Children in the NDD, EBP, and BOTH groups were more likely to be boys, live with a single parent, have a parent with less than or equal to high school education, and live in a low income household. Moreover, children in the NDD and BOTH groups were more likely to be school-age children as compared to children in the HEALTHY group. Consistent with our previous work [14, 38], these findings suggested some sociodemographic differences among the child health groups.

Regarding participation in sports or physical activity, children in the NDD, EBP, and BOTH groups were significantly less likely to participate in organized sports or physical activities than children in the HEALTHY group (see Table 1). This finding was particularly striking for the BOTH group; whereas more than 70% of children in the HEALTHY group participated in organized sports or physical activities at least once a week, only half of children in the BOTH group, and slightly more than half (55%) of the children in the NDD group participated in organized sports or physical activities at least once a week. This was followed by children in the EBP group (66%). In contrast, there were no statistically significant differences by health group for children's participation in unorganized sports or physical activities. It should be noted that because we did not find any statistically significant differences in children's participation in unorganized sports or physical activities, this variable was not analyzed further.

The results from the logistic regression analyses are summarized in Table 2. Consistent with our comparison analyses, the results from Model 1 indicated that children in the NDD, EBP, and BOTH groups were less likely to participate in organized sports or physical activities as compared to children in the HEALTHY group. The findings from the contrast analyses suggested that the independent effect of being in the NDD and BOTH groups was similar (Wald *F* = .50, *P* > .05), while the independent effect of being in the EBP group was significantly lower than that for children in the NDD (Wald *F* = 5.69, *P* < .05) and BOTH (Wald *F* = 7.80, *P* < .05) groups. These findings suggest that the association between children's participation in organized sports or physical activities is stronger for children in the NDD and BOTH group than the EBP group, with these children reporting significantly less participation in organized sports or physical activities as compared to children in the HEALTHY group.

The findings from Model 2 suggested that the associations between child health group and participation in organized sports or physical activities in Model 1 remained statistically significant even after controlling for the effects of child age and sex. As expected, child age, but not sex, was significantly associated with participation in organized sports or physical activities. That is, school-age children had twice the odds (OR = 2.09, *P* < .001) of participating in organized sports or physical activities as compared to preschool-age children. This finding suggests that the child's health condition (NDD, EBP, BOTH) is associated with organized sports or physical activities over and above the effect of child age.

The results from Model 3 indicated that children in the NDD and BOTH groups were less likely to participate in organized sports or physical activities at least once a week over and above the effects of other child and family sociodemographic characteristics, including parental education, household income, and the child living in a CMA or CA. Interestingly, child EBP condition was no longer significantly associated with participation in organized sports or physical activities (OR = .90, *P* > .05) when controlling for sociodemographic characteristics. Furthermore, school-age children (OR = 2.31, *P* < .001), children who have a parent with greater than high school education (OR = 2.48, *P* < .001), who live in a high income family (OR = 2.32, *P* < .001), and in a CMA or CA (OR = 1.39, *P* < .001) were more likely to participate in organized sports or physical activities at least once a week.

We conducted additional logistic regression analyses, where each variable was examined separately, to determine which factors mediated the relationship between child health condition and participation. The findings indicated that parent's educational attainment and household income played a role in mediating the association between child EBP condition and participation in organized sports or physical activities.

Overall, these findings indicate that by comparison children in the NDD and BOTH groups were less likely to participate in organized sports or physical activities even after controlling for the effects of other child and family sociodemographic characteristics. In contrast, the likelihood of participation in organized sports or physical activities for children in the EBP group was no longer statistically different than children in the HEALTHY group after accounting for the differences in other child and family sociodemographic characteristics. A similar pattern to the first set of contrast analyses was found. Specifically, the independent effect of being in the NDD and BOTH groups was similar (Wald *F* = .61, *P* > .05), while the independent effect of being in the EBP group was significantly lower than that for children in the NDD (Wald *F* = 7.96, *P* < .01) and BOTH (Wald *F* = 8.80, *P* < .01) groups. These findings suggest that, even after controlling for the effects of other child and family sociodemographic characteristics, the association between children's participation in organized sports or physical activities is stronger for children in the NDD and BOTH group, with these children reporting significantly less participation in organized sports or physical activities as compared to children in the HEALTHY group.

## 4. Discussion

This study used a population-based Canadian survey to examine the physical activity participation of children with NDDs with and without EBPs as compared to children without disability. The results revealed that children with both NDDs and EBPs, possibly representing more “complex” health challenges, were the least likely to participate in organized physical activity, followed by children with NDDs (although the NDD group did not differ significantly from the BOTH group) and then those with EBPs as compared to children without disability.

It is possible that children with varying degrees of disability have fewer opportunities or less time to participate in organized physical activity [3], or that children with complex disabilities are less likely to be capable or feel competent in organized activities. However, the fact that children in the comorbid NDD and EBP group as well as the NDD group participated less than children without disability supports previous research that has found a link between severity of condition and activity participation [25, 27]. Severity of the condition (including the presence of a co-morbid condition) may be particularly relevant in organized activity participation which requires formal motor skills and expertise, for example, the ability to manipulate a ball in many organized sports. Disability type has also previously been associated with children's perceptions of fitness, with those children with physical disabilities less likely to perceive themselves as fit as their peers than those with a chronic medical condition [26]. Perceptions of fitness may then translate into different selection of activities, including reduced participation in organized physical activities.

However, differences in physical activity participation were demonstrated for organized but not unorganized physical activities. For unorganized physical activities, the majority of each group participated and no differences in participation rates were found in comparison to children without disability. This is in line with previous research suggesting that children with CP are more likely to engage in informal, rather than formal, physical activities [23, 31, 32]. The findings might be explained by the fact that informal activities may be more easily adapted and rules more flexible to allow for increased participation so that children with disabilities are at less of a disadvantage than is the case for formal, organized, and perhaps competitive, physical activities. Certainly, environmental barriers such as lack of adequate space and adapted activities which accommodate children's needs may be less of a concern with informal (or unorganized) types of activities [32]. There are certainly differences that need to be considered concerning availability of unorganized physical activity. For example, in Canada, children tend to participate in privately organized programming (e.g., sports clubs and organizations); however, in the United States of America (USA), many children participate in privately and federally-funded after-school programming [46].

The findings from the present study also point to other important factors that have an impact on the physical activity participation of children with disabilities, namely, of family sociodemographics. Although parent sociodemographic factors were associated with physical activity participation, they did not mediate associations of NDDs with or without EBPs and physical activity, perhaps pointing to the importance of other characteristics such as severity and motor functioning. However, parental sociodemographic characteristics did have an impact on the likelihood of participation for children with EBPs. The likelihood of participation in organized physical activities for children with EBPs who were of school age, lived in a CMA or CA, and had parents with higher levels of education and income, was no different than for children without disability. It is beyond the scope of the present study to speculate on the reasons for this but factors such as living in a CMA or CA and parental socio-economic resources may allow for differences in opportunities, programs, and environments conducive to the participation of children with EPBs.

However, for children with an NDD and those with both an NDD and an EBP, parent sociodemographic characteristics did not fully mediate the child health effects on the probability of participation in organized physical activity. These findings suggest that lower levels of participation for children with an NDD and both an NDD and EBP were not explained by sociodemographic characteristics measured in the current study. Findings from other international studies have suggested possible explanatory factors regarding participation in physical activity for children with disabilities. For example, gross motor function was shown to be a significant predictor of overall physical activity for Australian youth (aged 11–17) with CP, with those with higher levels of gross motor impairment being less likely to participate [20]. In addition, other research has suggested that child and family preferences for activity, family factors (such as cohesion), and environmental conditions (including babysitting or transportation) may be directly or indirectly associated with physical activity participation for children with disabilities [6, 32, 34]. These measures were not included in the present study as they were not available in the NLSCY and thus could not be considered. Yet, it is important to realize that factors relating not only the child but also to the family and the environment are associated with participation in physical activities for children with disabilities.

Strengths of this study include the use of a population-based sample of children which allowed us to include children with NDDs both with and without EBPs as well as an age-matched comparison group of children who had no chronic conditions or activity limitations. Previous research has been limited by small, sometimes condition-specific samples [5], with a particular focus on children with CP. We were also able to report rates of participation separately for organized as well as unorganized activities. Despite these strengths, limitations of the study include limited information on the severity of the child's condition and motor functioning, availability of appropriate activities (e.g., adapted activities), and measures of the physical environment which may impact opportunities for participation [6, 32, 34]. Finally, although a national study, sample sizes were too small to examine other questions such as the interaction effects (e.g., between gender and child health group) on physical activity participation.

We were also somewhat limited by the measure of physical activity included in the NLSCY survey. Although parent or self-reported items are frequently used in national surveys collecting physical activity information, there are limitations associated with this type of data such as reporting bias and respondent definitions of organized and unorganized activities. In addition, the parent-reported measure of physical activity included in the NLSCY does not include information about intensity, with whom the activity was performed, or preferences for activity (as does the *Children's Assessment of Participation and Enjoyment* (CAPE)), which is used in many other studies of physical activity in children or youth with disabilities [5, 6]. 

## 5. Conclusions

These findings have implications for practice and research, both within Canada and internationally. Specifically, our results highlight the importance of considering children's primary and other existing health conditions as well as family sociodemographic characteristics in order to better understand the factors that influence participation in organized physical activities. Our results also suggest that the largest differences in participation for children with health conditions is for organized and not unorganized physical activities. Policies aimed at increasing participation rates of children with health conditions could focus on facilitating and increasing participation in organized physical activity and for some children such as those experiencing EBPs, ensuring that cost and availability are not barriers to access. While results from the current study suggest that Canadian children with an NDD and those with an NDD and an EBP are less likely to engage in organized physical activity than are their peers without disability, they are no less likely to engage in unorganized physical activity.

## Figures and Tables

**Table 1:** Descriptive statistics of all study variables.

Characteristics (%)	Overall *N* = 9119	NDD *n* = 286	EBP *n* = 1382	BOTH *n* = 137	HEALTHY *n* = 7314
Child is school-aged	67	83*	69	89*	66
Child is male	49	65*	53*	73*	48
Child lives with a single parent	14	24*	19*	24*	13
Parent's educational attainment (>high school)	72	61*	68*	54*	74
Household income					
Low income	15	24*	20*	28*	14
Moderate income	35	37	38*	27	34
High income	50	39*	42*	45	52
Child lives in a CMA or CA	81	82	80	83	81
Child participates in organized sports or physical activities at least once a week	70	55*	66*	50*	71
Child participates in unorganized sports or physical activities at least once a week	71	73	72	63	71

*The superscript indicates group differences, with the reference group being HEALTHY at the *P* < .05 level.

CMA: census metropolitan area. CA: census agglomeration.

Source: National Longitudinal Survey of Children and Youth 2006-2007, Statistics Canada.

**Table 2:** Summary of results from logistic regression analyses predicting children's participation in organized sports or physical activities (*N* = 9119).

Variables	Model 1		Model 2		Model 3	
	OR	95% C.I.	OR	95% C.I.	OR	95% C.I.
NDD	.49***	.34–.71	.42***	.29–.60	.50***	.34–.72
EBP	.79*	.66–.95	.76**	.64–.92	.90	.74–1.10
BOTH	.40***	.25–.63	.32***	.20–.51	.39***	.22–.67
HEALTHY	Ref		Ref		Ref	
Child is school-aged			2.09***	1.80–2.43	2.31***	1.96–2.73
Child is boy			1.06	.92–1.21	1.07	.92–1.24
Child lives with a single parent					1.03	.84–1.27
Parent's educational attainment (>high school)					2.48***	2.12–2.89
Household income						
Low income					.48***	.39–.60
Moderate income					Ref	
High income					2.32***	1.97–2.74
Child lives in a CMA or CA					1.39***	1.19–1.63

Ref: the reference group. CMA: census metropolitan area. CA: census agglomeration.

Note. All reported values are odd ratios (OR) with 95% confidence intervals (CI).

*P < .05. **P < .01. ***P < .001.

Source: National Longitudinal Survey of Children and Youth 2006-2007, Statistics Canada.
